# Evaluation of Mobile and Community Dental Service Use among People Experiencing Homelessness

**DOI:** 10.3390/ijerph20010845

**Published:** 2023-01-02

**Authors:** Bohuslav Novak, Marek Matajs, Alessandro Emanuele Sangalli, Halyna Pruts, Anna Korpasova, Nikos Leptos, Peter Stanko, Michal Tinak, Michaela Kosticova

**Affiliations:** 1 Unit of Conservative Dentistry, Department of Stomatology and Maxillofacial Surgery, Faculty of Medicine, Comenius University in Bratislava, Heydukova 10, 812 50 Bratislava, Slovakia; 2 Unit of Prosthetic Dentistry, Department of Stomatology and Maxillofacial Surgery, Faculty of Medicine, Comenius University in Bratislava, Heydukova 10, 812 50 Bratislava, Slovakia; 3 Unit of Oral and Maxillofacial Surgery, Department of Stomatology and Maxillofacial Surgery, Faculty of Medicine, Comenius University in Bratislava, Heydukova 10, 812 50 Bratislava, Slovakia; 4 Faculty of Medicine, Comenius University in Bratislava, Spitalska 24, 813 72 Bratislava, Slovakia; 5 Institute of Social Medicine and Medical Ethics, Faculty of Medicine, Comenius University in Bratislava, Sasinkova 2, 813 72 Bratislava, Slovakia

**Keywords:** homeless persons, dental care, community health service, access to care

## Abstract

The aim of the study was to evaluate the patterns of mobile and community dental service use among people experiencing homelessness in Bratislava, Slovakia. Data from medical records of homeless people who visited the mobile and community dental clinic from November 2012 to July 2018 have been retrospectively reviewed. The experience of providing dental services has been reported from the perspective of the dental care provider. Descriptive statistics were used for data analysis. In total, 319 patients (75.5% men) attended the clinic. Extraction of a tooth was the main dental treatment indicated in more than 80% (n = 276) of patients. A total of 363 teeth were extracted with an average number of 1.6 extracted teeth per patient. The main indications for extraction were dental caries and its sequelae (83.7%) and periodontal disease (15.2%). The patients with the need for prosthetic and conservative treatment were referred to the clinics at University Hospital; however, only 19 patients received the treatment. The barriers to accessing dental care were cost, logistic problems and individual psychological factors. We found that people experiencing homelessness in Slovakia have high needs and demands of emergency dental care and many systemic and individual barriers prevent them from accessing care. Community-based dental services are important for improving access to dental care and reducing oral health inequities.

## 1. Introduction

Homelessness represents an extreme form of vulnerability, poverty and social exclusion, contributes to significant morbidity, disability, premature mortality, increased healthcare utilization rates and is the cause of health inequities [1,2,3]. Based on census data, more than 20,000 people experiencing homelessness lived in Slovakia in 2011 and more than 2000 of them (64.5% men) were in the capital city of Bratislava [4]. Regarding oral health, homeless people experience worse oral health and poorer access to dental services than the general population [5,6]. They have more untreated dental caries, more severe tooth loss, suffered from more gum diseases and were more likely to experience toothache and dental trauma than the general population [6,7,8,9]. A range of factors contribute to the limited access to dental care, including patient-related factors, systemic factors and dental-care-professional-related factors [5,8,10,11]. There is evidence that homeless people often go to an emergency department for their dental and other health problems [2,9,12]. Emergency care is the only care most people experiencing homelessness in Slovakia are eligible to receive because of having debts on health insurance payments [13]. The evidence shows that community-based oral health interventions are crucial for improving the access of homeless people to dental care [10,11,14,15,16,17]; however, such services have not been developed in Slovakia yet. The only community-based dental clinic providing emergency dental care for people experiencing homelessness was open from 2012 until 2018 in Bratislava. The aim of our report was to evaluate the patterns of dental service use among homeless people visiting this clinic and present the lessons learned from this unique five-year project.

## 2. Materials and Methods

Data from the medical records of homeless people who visited the mobile and community dental clinic in Bratislava from the 1st of November 2012 to the 31st of July 2018 have been retrospectively reviewed to evaluate the patterns of dental service use and map the dental treatment needs and demands of homeless people. The experience and lessons learned from the mobile and community dental clinic project have been reported from the perspective of the dental surgeon who established the clinic and provided the dental care.

The following data have been analyzed: gender, age, comorbidities considered as a risk for the provision of dental care, type of tooth/teeth removed, indications for the extraction, number of visits, referrals and type of further dental treatment. The comorbidities were self-reported by patients to the dentist during a dental examination. The indications for extraction were categorized in four main groups: (A) dental caries and its sequelae, (B) localized or generalized periodontal disease with tooth/teeth mobility of grade two or three according to Grace and Smales mobility index [18], (C) trauma (dental or jaw fracture) and (D)other indications (e.g., partially impacted tooth, recurrent pericoronitis, etc.). Group A was divided into three subgroups as follows [19]: irreversible pulpitis (spontaneous radiating pain without any swelling), acute periostitis of the upper or lower jaw (pain and extraoral swelling) and dental roots (teeth severely damaged by caries or retained roots without pain or swelling). Descriptive statistics were used to characterize the studied group of patients and patterns of dental service use.

## 3. Results

### 3.1. Description of Dental Services

The mobile dental clinic was opened as a part of the Mobile Ambulance Station (MAS) in Bratislava in November 2012. Seven volunteers (one general practitioner, one oral surgeon, three medical students and two nurses) from three organizations: Monastic Society, the Brothers of Saint John of God, the Organization Maltese Help and the University Hospital in Bratislava provided emergency dental and health care for people experiencing homelessness. Extraction of teeth was the main dental treatment procedure provided. The MAS was located in the city center and opened once a week in the evening. The MAS project finished in December 2013 and continued as a community dental clinic in a low-threshold day center for homeless people located at the city’s periphery. One dental surgeon and one dentistry student provided emergency dental care for free once a week. Due to the limited financial and material resources, only extractions of teeth were provided; patients who needed conservative dental treatment or prosthetics were referred to specialized units at the University Hospital, Department of Stomatology and Maxillofacial Surgery (DS&MFS). The community dental clinic was closed in July 2018 due to the retirement of the oral surgeon and problems with human and material resources. Currently, no community-based dental services are available for people experiencing homelessness in Bratislava.

### 3.2. Characteristics of Patients and Type of Dental Services

The characteristics of patients and types of dental services used are presented in Table 1. In total, 319 patients (241, 75.5% men) visited the mobile and community dental clinic from the 1st of November 2012 to the 31st of July 2018. The age range of the patients was 18–86 years, mean age 43.3 years (males 45.5; females 41.1). The total number of visits was 614 (1.83 visits/one man: 2.2 visits/one woman). Extraction of tooth/teeth was the main dental treatment indicated in more than 80% (n = 276) of patients. The follow-up dental check-ups were provided for 140 (58.1%) men and 48 (61.5%) women.

#### 3.2.1. Comorbidity Analysis

Patients with self-reported comorbidities, which might be considered as a risk for the provision of dental care, accounted for only 16.3% (n = 52). More women (23%) than men (14.1%) reported some kind of health problem. Eleven patients reported previous infectious diseases including hepatitis C, hepatitis B and HIV positivity. Ten patients reported cardiovascular diseases including three past myocardial infarctions and seven other cardiovascular pathologies. Other comorbidities reported by patients were neurological disorders (n = 7) including epilepsy, stroke and multiple sclerosis, diabetes mellitus (n = 4), bronchial asthma (n = 3), cancer (n = 5), drug abuse (n = 4), mental disorders (n = 3) and drug allergy (n = 5). However, no medical documentation was available to support the reported data about the comorbidities. Dental care was provided to three pregnant women.

#### 3.2.2. Follow-Up Dental Treatment

In total, 29 patients received specialized or follow-up dental treatment at the DS&MFS. Four patients with complications (e.g., root fracture, oroantral fistula) and six risk-bearing patients were referred to DS&MFS for specialized dental care. Outpatient surgical treatment was provided to six men and four patients required hospitalization. Follow-up conservative treatment was realized only in seven patients with dental caries and endodontics by dentistry students of the Faculty of Medicine, Comenius University in Bratislava, under the supervision of their teachers. Further prosthetics were continued for 12 patients. Patients should order themselves on the provided phone contact and had to pay for the treatment. The reasons, reported by patients to community dentists, why most of them did not receive the care were the cost strains, dental fear, embarrassment about the conditions of their teeth, being afraid of stigmatization, logistic and communication problems with the arrangement of the appointments and refusal of the conservative treatment.

### 3.3. Teeth Extractions Analysis

Characteristics of the teeth extractions are presented in Table 2. A total of 363 teeth (271, 74.7% in men) were extracted. The average number of extracted teeth was 1.14 per patient in total and 1.6 per patient with extractions. Most extractions were in the lower jaw (n = 200, 55.0%) and on the right side. Molars accounted for 51.5 % (n = 187) of the teeth removed, with the dominancy of the second right lower molars. The percentage of extracted premolars and anteriors appeared at a similar level (23.2%/25.3%). The main indication for extraction was dental caries and its sequelae (n = 304, 83.7%). In the subgroup analysis, the most frequent diagnosis was dental roots (n = 210, 57.9%), followed by pulpitis (n = 73, 20.1%) and periostitis (n = 21, 5.8%). The second most frequent indication for the extraction was periodontal disease with tooth mobility of grade 2 or 3 (n = 55, 15.2%). The trauma group included three cases (0.8%) and one extraction was performed in a woman due to recurrent pericoronitis of the lower wisdom tooth. Four patients refused the indicated extraction and the extractions were not realized in two drunk men.

## 4. Discussion

This is the first study mapping the patterns of mobile and community dental service use among people experiencing homelessness in Slovakia and central Europe. We found that people experiencing homelessness have high needs and demands for emergency dental care and a lot of barriers on systemic and individual levels prevent them from accessing the care. Mobile and community dental clinics are the first contact points with dentistry and often the only services accessible for people experiencing homelessness. Our experience suggests they are positively perceived by patients and healthcare providers; however, a lot of problems exist in relation to their existence, the range of services provided and sustainability.

In total, 319 homeless people, mostly middle-aged men with acute dental problems, visited the mobile and community dental clinic. The extraction of teeth was the main treatment procedure provided. This was mainly because patients suffered from serious dental problems that could not be restored with conservative treatment; moreover, some patients preferred the extraction of teeth to the conservative treatment and finally the dental clinic had no equipment and materials to provide the conservative treatment. The main indication for the extraction was dental caries (83.7%) and its sequelae, followed by periodontal diseases (15.2%), with the dominant location on molars in the lower jaw. The indications for extraction were the same as for the general population. However, the prevalence of extractions in people experiencing homelessness was almost eight times higher than in the general population in Slovakia. According to the routinely reported national data on dental care, teeth extractions were provided only in 11.2% of patients who visited dental clinics in Slovakia from 2013 to 2018, compared with 86.5% of homeless patients who visited the community dental clinic [19]. Our results correspond with the results of the studies from developing countries [20,21]; however, no data are available from central European countries.

Patients with comorbidities accounted only for 16.3% (n = 52) in our study and only six patients required specialized care in the hospital. The most frequently reported diseases were infectious diseases (Hepatitis B, C and HIV infection) and cardiovascular diseases. Just a few patients reported mental health problems and drug abuse. The evidence from other studies shows a higher prevalence of infectious diseases, cardiovascular diseases, mental disorders and substance abuse in the population of people experiencing homelessness [3,22,23,24]. However, based on our experience and reported also by Daly et al., the medical conditions of homeless people had little impact on their dental care and most dental care could be provided in the community or primary-care settings [25].

The patients with the need for conservative or prosthetic treatment were referred to the dental clinic at the University Hospital. However, most of them did not receive the treatment because of not being able to pay for the treatment, feeling ashamed or anxious, having dental fear and also because of logistic and communication problems. These findings are in line with the results of the studies from other countries such as the United Kingdom [10,15,16,26], Canada [27], Italy [17] and Australia [11,28], which pointed out that the main barriers limiting the access of people experiencing homelessness to dental care originated from the health care system itself, as it was not adapted to the complex needs of the homeless population. Based on the evidence from these studies and our experience, a successful dental service needs to be informal, flexible, adapted to the complex needs of homeless persons and provided for free in the community where people live. Although we found a high demand from people experiencing homelessness for a community dental service, the problems with financial, material and human resources and administrative and legislation problems were the barriers to continuing and sustaining providing the service. Evidence suggests that collaboration with key stakeholders and end-users, including co-design of the services with homeless people; multidisciplinary working with homeless support organizations, dental practitioners, and educational institutions; and public funding are crucial to enhance the acceptability, feasibility and sustainability of community-based dental services [10,11,14,28]. Moreover, based on the evidence, the community-based participatory approach in research is important not only to increase the likelihood of community-based intervention success but also to eliminate oral health disparities [29,30].

### 4.1. Limitations and Strenghts

There are some limitations in the study. First, the retrospective design of the study and unavailable medical documentation limits the interpretation of the data. Second, the evaluation of the service use was presented from the dental provider’s perspective. Perceptions of patients were not included, so their opinions on the fulfillment of their dental care needs and demands might be different. Third, the dental services were provided just once a week with limited dental equipment and material resources; thus, the extraction of teeth was the main treatment procedure provided and the patients with conservative treatment needs were sent to the clinics at the University Hospital without any data collected. Therefore, the data about conservative treatment might be underestimated in our study. Finally, selection bias needs to be considered when interpreting results. Although the sample is not representative of all people experiencing homelessness in Slovakia, it is representative of those who used the services in the dental clinic over the five years in Bratislava. However, the findings cannot be generalized to all homeless people in Bratislava due to the physical, psychological and social barriers that prevented some people from visiting the clinic. Moreover, the transferability of our findings to other countries may be difficult due to differences in the organization and financing of dental care.

Despite its limitations, the study brings valuable information about the dental needs of a hard-to-reach vulnerable population. The strength of the study is its community-based participatory approach with the engagement of dental care providers, the community and academic members. Furthermore, the study might be seen as an example of good practice and the first step in the development of community-based dental services in Slovakia.

### 4.2. Implications

Our report pointed out that community-based dental services are important to address the urgent dental needs of people experiencing homelessness. However, to tackle the oral health inequities, more complex actions are needed, including changes in the health system, the development of integrated health care and actions toward the broader social determinants of health. Regarding the health system in Slovakia, the changes in legislation, organization and financing of health care are required to enable the development of community-based health services and ensure access to dental and health care for socially excluded populations with debts on health insurance payments. Furthermore, examples from other countries showed that new flexible mixed models of private and public funding need to be developed to ensure the sustainability of providing care for socially vulnerable populations with high dental needs [16].

Future research should be conducted with a larger sample of homeless patients and in collaboration with other community health providers who could identify patients with dental treatment needs and provide more objective data about their health status. In order to obtain more data about conservative and prosthetic treatment, the collaboration with dental providers at the University hospital needs to be enhanced. Moreover, additional data on socio-demographic and psychosocial factors that might be related to the patterns of use of dental care among people experiencing homelessness should be collected. Conducting interviews with homeless people would help to explore the factors in more detail. Furthermore, interviews with dental care providers and policymakers could bring more information about their views and opinions regarding the challenges of providing dental care for people experiencing homelessness and identify the enablers for the effective and sustainable provision of community dental services.

## 5. Conclusions

Our findings have shown that outreached and community-based dental services are crucial for improving the access of people experiencing homelessness to dental care and should be provided in collaboration with homeless support organizations, other health care providers and educational institutions to address the complex needs of homeless people. Moreover, we argue that a mixed model of private and public funding of community dental services is needed to ensure their sustainability. Finally, we suggest that effective strategies and interventions to improve oral health equity should focus on both downstream and upstream determinants of oral health.

## Figures and Tables

**Table 1:** Characteristics of patients (homeless people, n = 319) and type of dental services use stratified by gender in Bratislava, Slovakia, 2012–2018.

Characteristic	Men (n = 241)	Women (n = 78)
	Frequency (%)	Frequency (%)
Age (years)		
Young age (18–39)	76 (31.5)	36 (46.2)
Middle age (40–59)	142 (58.9)	39 (50.0)
Old age (60+)	23 (9.5)	3 (3.8)
Visits		
Number of visits	442 (1.83 visits/1 man)	172 (2.2 visits/1 woman)
Single check-ups	101 (41.9)	30 (38.5)
Follow up check-ups	140 (58.1)	48 (61.5)
Type of dental treatment		
Extractions	213 (88.4)	63 (80.8)
Dental care without extraction	28 (11.6)	15 (19.2)
Follow-up dental treatment at University Hospital, DS&MFS ^1^	17 (7.0)	12 (15.38)
Outpatient surgical treatment	5	1
Inpatient dental care	3	1
Prosthetic dental care	4	8
Outpatient conservative treatment	5	2
Comorbidities	34 (14.1)	18 (23.0)
Infectious (HIV, HBV, HCV)	7	4
Cardiovascular	8	2
Neurological	6	1
Cancer	1	4
Drug allergy	3	2
Diabetes	3	1
Drug abuse	2	2
Mental disorders	1	2
Bronchial asthma	3	0
Gravidity		3

^1^ Department of Stomatology and Maxillofacial Surgery.

**Table 2:** Characteristics of the teeth extractions (n = 363) stratified by gender, indication and location; homeless people (n = 319), Bratislava, Slovakia, 2012–2018.

Characteristic	Teeth Extractions
	Proportion (%)
Gender	
Men	271 (74.7)
Women	92 (25.3)
Indication	
(A) Dental caries and its sequelae	304 (83.7)
Radix dentis	210 (57.9)
Pulpitis dentis	73 (20.1)
Periodontitis apicalis acuta	21 (5.8)
(B) Periodontal disease (tooth mobility grade 2 or 3)	55 (15.2)
(C) Fractura dentis traumatica	3 (0.80)
(D) Other indications (*recurrent pericoronitis)*	1 (0.20)
Location	
Mandible	200 (55.0)
Lower anteriors	44 (12.0)
Lower premolars	47 (13.0)
Lower molars	109 (30.0)
Maxilla	163 (45.0)
Upper anteriors	48 (13.3)
Upper premolars	37 (10.2)
Upper molars	78 (21.5)
Refused extractions	6

## Data Availability

The data presented in this study are available on request from the corresponding author. The data are not publicly available due to ethical restrictions.
